# Etymologia: *Enterocytozoon bieneusi*

**DOI:** 10.3201/eid2706.ET2706

**Published:** 2021-06

**Authors:** Maxime Moniot, Philippe Poirier, Céline Nourrisson

**Affiliations:** Centre Hospitalier Universitaire, Clermont-Ferrand, France

**Keywords:** Enterocytozoon bieneusi, fungi, parasites, obligate intracellular parasites, microsporidia, microsporidiosis, HIV/AIDS pandemic

## *Enterocytozoon bieneusi* [′entərəˌsaitə′ӡu:ən bıə′nəʊsı]

From the Greek *énteron* (intestine), *kútos* (vessel, cell), and *zỗion* (animal), and the surname Bieneus, in memory of the first infected patient whose case was reported in Haiti during 1985. *Enterocytozoon bieneusi*, a member of the wide-ranging phylum Microsporidia, is the only species of this genus known to infect humans. Microsporidia are unicellular intracellular parasites closely related to fungi, although the nature of the relationship is not clear (Figure).

**Figure:**
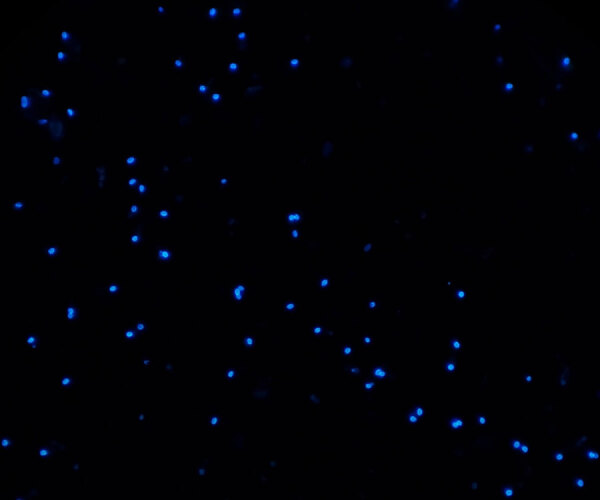
Spores of *Enterocytozoon bieneusi* in a fecal smear from a patient with intestinal microsporidiosis. Spores are small (≈1.5 µm × 0.5 µm) and egg-shaped (calcofluor-white stain, original magnification ×1,000). Photograph courtesy of the corresponding author.

*E. bieneusi*, a spore-forming, obligate intracellular eukaryote, was discovered during the HIV/AIDS pandemic and is the main species responsible for intestinal microsporidiosis, a lethal disease before widespread use of antiretroviral therapies. More than 500 genotypes are described, which are divided into different host-specific or zoonotic groups. This pathogen is an emerging issue in solid organ transplantation, especially in renal transplant recipients.

## References

[1] Desportes I, Le Charpentier Y, Galian A, Bernard F, Cochand-Priollet B, Lavergne A, et al. Occurrence of a new microsporidan: *Enterocytozoon bieneusi* n.g., n. sp., in the enterocytes of a human patient with AIDS. J Protozool. 1985;32:250–4. 10.1111/j.1550-7408.1985.tb03046.x 4009510

[2] Didier ES, Weiss LM. Microsporidiosis: not just in AIDS patients. Curr Opin Infect Dis. 2011;24:490–5. 10.1097/QCO.0b013e32834aa152 21844802 PMC3416021

[3] Han B, Weiss LM. Microsporidia: obligate intracellular pathogens within the fungal kingdom. Microbiol Spectr. 2017;5:97–113. 28944750 10.1128/microbiolspec.funk-0018-2016 PMC5613672

[4] Moniot M, Nourrisson C, Faure C, Delbac F, Favennec L, Dalle F, et al. Assessment of a multiplex PCR for the simultaneous diagnosis of intestinal cryptosporidiosis and microsporidiosis: epidemiologic report from a French prospective study. J Mol Diagn. 2021;23:417–23. 10.1016/j.jmoldx.2020.12.005 33387699

